# Effect of artificial sweat, pigmentation and adhesives for fixing facial prostheses on physical and optical properties of two facial silicones

**DOI:** 10.2340/biid.v12.44660

**Published:** 2025-09-25

**Authors:** Gabriele Martins, Daniela Micheline dos Santos, Bárbara Luise Medeiros dos Santos, Fernanda Pereira de Caxias, Marcelo Coelho Goiato

**Affiliations:** a Department of Dental Materials and Prosthodontics, Araçatuba Dental School, São Paulo State University (UNESP), São Paulo, Brazil

**Keywords:** facial silicone, adhesives, pigmentation

## Abstract

The aim of this study was to evaluate the effect of sweat and adhesives on color stability, roughness (Ra) and Shore A hardness of two silicones for facial prostheses with different pigmentation. Samples of Silastic MDX4-4210 and A-2186 silicone were made for the proposed tests, distributed into 12 groups according to their pigmentation and adhesive used and were immersed in artificial sweat for 3 months, disinfected every 3 days with neutral soap. Measurements of color stability, roughness and hardness were performed according to ISO 21920 and CIEDE2000 using a spectrophotometer and ISO 868, respectively. There was a statistically significant color change in the two silicones used, with the groups that had the least change being those without adhesive applied to their surface (controls). There was also a reduction in roughness in all groups, with MDX4-4210 being the roughest silicone. In terms of Shore A hardness, all the groups became harder after the experimental period, with A-2186 achieving the highest results. Color change, Shore A hardness and roughness showed significant changes in the MDX4-4210 and A-2186 silicones for the proposed tests. Even so, all the results found were clinically acceptable, making both silicones excellent options for use in maxillofacial rehabilitation.

## Introduction

Oral and maxillofacial prosthetics is a humanitarian dental specialty of great importance for patients with orofacial defects, since the prosthetic restoration of facial deformities allows these patients to resume an active role in society [1].

Facial prostheses can be made using polymethyl methacrylate (PMMA) or silicone elastomers [2]. Studies in literature report that silicone elastomer is the most widely used option for making maxillofacial prostheses [3, 4], since in addition to being biocompatible [5–7], it is relatively inexpensive, can be characterized and pigmented [3], has a texture similar to the skin and physical [5, 7–9] and mechanical [9, 10] properties conducive to its use.

Typically, maxillofacial prostheses require replacement within a year due to fading of their color [3]. The success of the aesthetic result achieved, and the imperceptibility of the prosthetic reconstruction are determined by the nature and extent of the injury, the manual skill and anatomical knowledge of the specialist and the properties of the material used to make the facial prosthesis [11].

Literature shows that studies of the mechanical properties of silicone elastomers for facial prostheses have been the subject of several studies [12–19]. They have investigated hardness, roughness [13], tensile strength and modulus, elongation, tear strength, hardness, weight and color change [12], and properties of adhesives used for the prosthesis’s fixation [16–19]. These studies were performed in situ [13] or in simulated clinical conditions [12, 14, 15]. The hardness of silicone determines its flexibility, and simulating the flexibility of human skin as faithfully as possible promotes greater comfort, aesthetics and functionality of the prosthesis for the patient [20]. Silicones’ optical and mechanical properties can be influenced by exogenous and endogenous factors such as oil, sweat, use of skin products and cleaning protocol [13, 14].

A high surface roughness implies a greater accumulation of microorganisms, influenced by the length of time the prosthesis has been stored. Therefore, to avoid infections, it is necessary to use a chemical disinfection material when handling prostheses, such as washing with neutral pH soap and water [21].

Maxillofacial prostheses are subject to variations in humidity, such as contact with sweat, a factor that can degrade silicone over time [3, 4]. Studies show that humidity and precipitation have a greater effect on pigmented silicone elastomer than heat and sunlight [2]. In addition, changes in its physical and mechanical properties can occur with the application of pigment [9, 20, 21, 22, 23], where dimensional instability due to deterioration of the material leads to maladaptation of the prosthesis [10].

One of the biggest limitations of this material is the difficulty of adhesion [23], so the use of adhesive systems or primers is essential to improve the adhesion of silicone prostheses to human skin. These materials are applied to the inner surface of the prosthesis and inserted into the skin, keeping the bond more stable and secure [2, 23]. Literature shows that adhesive properties change along with the use and contact with skin [17].

The aim of this study was to evaluate the effect of sweat and adhesives on color stability, roughness (Ra) and Shore A hardness of two silicones for facial prostheses with different pigmentation. The null hypothesis was that there would be no statistical difference in color change, Shore A hardness and roughness between silicones MDX4-4210 and A-2186, under the influence of pigments, adhesives for fixation and artificial sweat.

## Materials and methods

The following materials were used to make the samples: Silastic MDX4-4210 (Dow Corning Corporation Medical Products, Midland, MI, USA) and A-2186 Silicone Elastomer (Factor II, Inc.) for maxillofacial prostheses (Lakeside, AZ, USA), artificial sweat (0.5% sodium chloride, 0.5% disodium phosphate and 0.05% histidine L in 1,000 mL of distilled water), Drying Adhesive (Custom DME, LLC, North South Lake, USA) and Pros-Aide Adhesive (A.D.M. Tronics, Inc., Northvale, USA), suitable for maxillofacial prostheses and the Factor pigments Factor (Tan FI – 215, Factor II, Inc., Lakeside, USA) and ‘Flocking’ (Beige, Dimclay, São Paulo, Brazil), as described in Table 1.

**Table 1:** Description of materials used for the sample fabrication.

Trademark	Manufacturer
Silastic MDX4-4210 Elastomer	Dow Corning Corporation Medical Products, Midland, USA
A-2186 Silicone Elastomer	Factor II, Inc., Lakeside, AZ, USA
Pigment Factor	Tan FI – 215, Factor II, Inc., Lakeside, USA
Pigment ‘*Flocking*’	Bege, Dimclay, São Paulo, Brazil
Artificial sweat	Farma Fórmula, Araçatuba, Brazil
Drying Adhesive	Custom DME, LLC, North South Lake, USA
Pros-Aide Adhesive	A.D.M. Tronics, Inc., Northvale, USA

Artificial ‘sweat’ for professional use refers to an aqueous solution that simulates the composition of human sweat. It has been obtained from pharmaceutical manipulation, prepared according to the International Standard Organization (ISO105-E04-2008E) [19], shown in Table 2. The pH was adjusted with NaOH or HCl [20].

**Table 2:** Components of artificial sweat.

Component	Concentration/Volume	
	ISO pH 5.5	ISSO pH 8.0
Sodium chloride (NaCl)	0.5%	0.5%
Disodium phosphate	0.5%	
Disodium hydrogen orthophosphate dodecahydrate		0.5%
Sodium dihydrogen orthophosphate dihydrate (Na2HPO4•12H2O)	0.22%	
L-histidine monohydrochloride monohydrate (C6H9O2N3•HCl•H2O)	0.05%	0.05%
Distilled water	1,000 mL	

The sequence of specimens was allocated into groups and predetermined using a randomization chart, possibly maintaining a degree of blinding of the samples.

**Figure 1:**
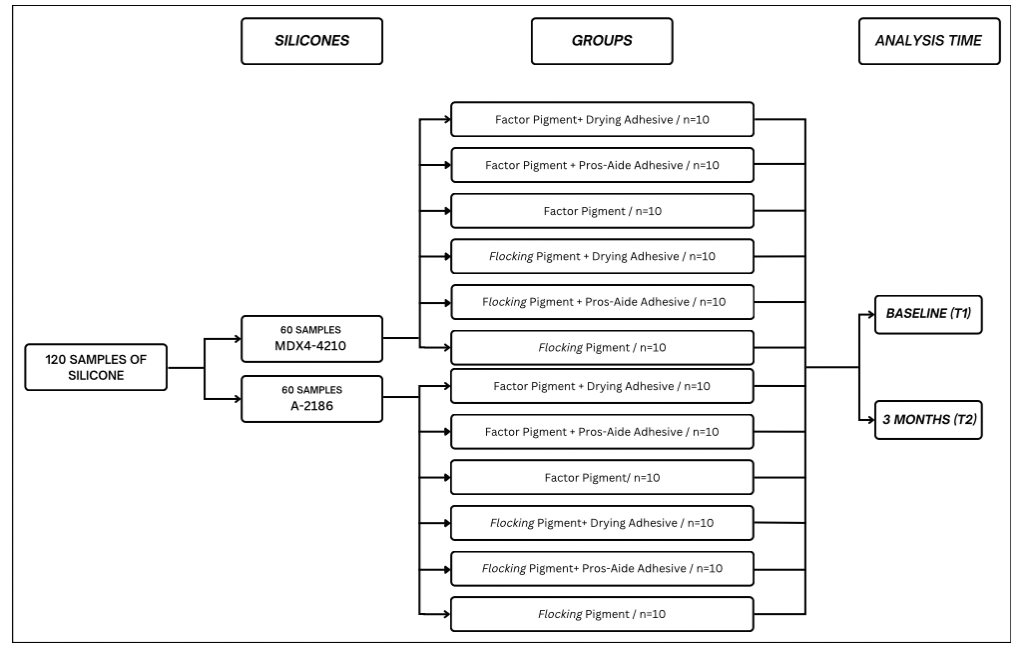
Distribution of samples according to group and proposed analysis time.

One hundred and twenty samples (*n* = 120) were made in a prefabricated metal matrix with 10 circular compartments measuring 10 mm(Ø) × 3 mm each for the Shore A hardness, surface roughness (Ra and Rt) and color change tests. Of these, 60 samples (*n* = 60) were made with Silastic MDX4-4210 silicone (30 samples with Factor pigment and 30 with Flocking pigment) and the remaining 60 with A-2186 Silicone Elastomer (30 samples with Factor pigment and 30 with Flocking pigment). These samples were divided into 12 groups of 10 samples each according to their pigmentation and the adhesive that would later be applied to their surface. The flowchart is represented in Figure 1.

The silicones were handled according to the manufacturer’s instructions at a controlled temperature of 23º ± 2ºC and relative humidity of 50 ± 10%. The pigments were weighed using an analytical balance (BEL Analytical Equipment, Brazil) and incorporated into the silicone using a stainless-steel spatula until they formed a homogeneous mass, corresponding to 0.2% of the weight of the silicone [21]. After handling, the silicones were inserted into the metal matrix and subjected to pressure for 24 hours in a pneumatic polymerizing pan (Metal Vander, SP, Brazil) to avoid the appearance of bubbles in the material. They were then confined inside the matrix with the outer surface exposed to the environment for 48 hours for complete polymerization of the material [24].

After they were made, initial color, surface roughness and Shore A hardness readings were taken, and all the samples were stored in artificial sweat manipulated by pharmacists in a bacteriological oven at 37 ± 1°C (CIENLAB Equipamentos Científicos Ltda, Campinas, SP, Brazil) [22] for 3 months to simulate the effect of sweat on silicone facial prostheses influenced by different types of pigments and adhesive systems over time. During this storage and ageing process, the specimens were cleaned every 3 days with neutral pH soap (Karité, Delicious Care, Dove, Brazil) and water, and the samples that were in contact with the adhesive were renewed with a new layer. At the end of the 3 months, the final color, roughness and Shore A hardness readings of the samples were taken for statistical analysis.

Color was analyzed according to CIE/ISO new standard: CIEDE2000 using a spectrophotometer. The color changes (ΔE) were calculated using the CIELab system, established by the Commission Internationale de I’Eclairage [18]. This system can be calculated according to the formula: ΔE = [(ΔL)2 + (Δa)2 + (Δb)2]½ 15. The ‘L’ represents the brightness from 0 (black) to 100 (perfect white), the ‘a’ represents the amount of red (positive values) or green (negative values) and the ‘b’ represents the amount of yellow (positive values) or blue (negative values) [24]. For this process, the Ultraviolet-Visible Reflectance Spectrophotometer (UV-2450, Shimadzu, Japan) was used.

Surface roughness was analyzed according to ISO 21920, using contact profilometry, using an SJ-401 surface roughness profilometer (Mitutoyo, Kanagawa, Japan), which has a 2 mm diameter diamond. The settings defined were: λ = 0.08 mm cutting wavelength and 0.25 mm transverse length at a speed of 0.05 mm/s for the surface roughness characteristics Ra and Rt. Ra is the average roughness which is determined by the arithmetic mean of the absolute values of the ordinates of the roughness profile. Rt is the roughness depth that represents the maximum peak height in the measured profile. Three measurements were taken on each sample, with the average defined as the roughness value [23].

Hardness was analyzed according to ISO 868. The hardness test (Shore A) samples were read using a durometer model GDS 709 (Teclock, Osaka, Japan) under the American Society for Testing and Materials (ASTM) specifications D-2240 [20, 23, 25, 26]. This method is based on the penetration of a needle into the surface of the material with a constant load of 10 N [20]. The measurement was set between 0 and 100 Shore A with a 1% tolerance. Each sample was placed in the durometer at a distance of 2 mm from the device’s penetration needle [20]. The needle applied pressure to the samples for 15 seconds and three measurements were made on each one, so that the three values obtained could then be averaged.

### Statistical analysis

The sample size calculation was performed retrospectively based on the observed effect size for the variable ‘ΔE’ (color change), which presented an F value of 10.590 in the analysis of variance (ANOVA) with 12 groups. The estimated effect size was *f* = 1.04 (a very large effect). Considering a significance level of 5% (α = 0.05) and statistical power of 80 and 90%, the minimum required sample size would be 3 specimens per group (total *n* = 36). As this study included 10 specimens per group (total *n* = 120), the sample size was more than sufficient to ensure statistical robustness.

Statistics were performed using Jamovi Software (Version 2.2.5.0, Jamovi Project, Australia). The tests performed were ANOVA with repeated measures, followed by the Tukey Honestly Significant Difference (HSD) test with a significance level of 5%. The evaluation of Color Chance was analyzed by Three-way ANOVA (Silicone × Pigment × Adhesive); Roughness tests were analyzed by four-way ANOVA (Time × Silicone × Pigment × Adhesive); Shore A hardness was analyzed by four-way ANOVA (Time × Silicone × Pigment × Adhesive).

## Results

Concerning color change (ΔE), the three-way ANOVA showed a statistically significant change in the color change values (Table 3) only between the adhesives (*p < 0.001). As shown in Table 4, the control groups had the significantly least color change for both silicones.

**Table 3:** Analysis of variance (three-way ANOVA repeated measures) of color change values.

Factors	Sum of squares	df	Mean square	*F*	*p*
Silicone	0.0505	1	0.0505	0.348	0.557
Pigment	0.3370	1	0.3370	2.321	0.131
Adhesive	3.0749	2	1.5374	10.590	< 0.001
Silicone × Pigment	0.2610	1	0.2610	1.798	0.183
Silicone × Adhesive	0.4283	2	0.2141	1.475	0.233
Pigment × Adhesive	0.7056	2	0.3528	2.430	0.093
Silicone × Pigment × Adhesive	0.3728	2	0.1864	1.284	0.281
Residuals	15.6799	108	0.1452		

**Table 4:** Mean values and standard deviation of the color change between the different silicones and adhesives used.

Silicones	DELTA 00 (ΔE)		
	Drying Adhesive	Pros-Aide Adhesive	Control
A-2186	1.77 A,a (0.267)	1.76 A,a (0.303)	1.43 A,b (0.317)
MDX-4210	1.8 A,a (0.424)	1.9 A,a (0.482)	1.5 A,b (90.42)

* *p* < 0.05 indicates statistically significant difference.

Averages followed by the same uppercase letter in the column and lowercase letter in the row do not differ at the 5% significance level (*P* < 0.05) by Tukey’s test.

Concerning roughness, the ANOVA analysis shown in Table 5 demonstrates that there was a statistically significant change (*p < 0.001) only to the time elapsed, but that the use of pigments and adhesives did not significantly alter the results. Furthermore, the statistical analysis showed a reduction in values after 3 months of immersion and levage (T2) for both silicones, regardless of the adhesive, with the MDX4-420 silicone having the highest roughness values, before and after the experimental period, compared to the A-2186 silicone (Table 6).

**Table 5:** Analysis of variance (four-way ANOVA repeated measures) of roughness values.

Factors	Sum of squares	df	Mean square	*F*	p
Time	2.05165	1	2.05165	194.192	< 0.001
Time × Silicone	0.09560	1	0.09560	9.049	0.003
Time × Pigment	0.00693	1	0.00693	0.656	0.420
Time × Adhesive	0.03077	2	0.01539	1.456	0.238
Time × Silicone × Pigment	0.01785	1	0.01785	1.690	0.196
Time × Silicone × Adhesive	0.02644	2	0.01322	1.251	0.290
Time × Pigment × Adhesive	0.00541	2	0.00270	0.256	0.775
Time × Silicone × Pigment × Adhesive	0.01036	2	0.00518	0.490	0.614
Residual	1.14102	108	0.01057		

**Table 6:** Mean values and standard deviation of roughness (Ra) between the different silicones and pigments used.

Silicones	Pigments	Time points	
		Initial (T1)	Final (T2)
A-2186	Factor (Tan FI – 215)	0.45 A,a (0.078)	0.28 A,b (0.062)
	Flocking	0.44 A,a (0.064)	0.32 AB,b (0.064)
MDX4-4210	Factor (Tan FI – 215)	0.57 B,a (0.156)	0.35 B,b (0.102)
	Flocking	0.62 B,a (0.168)	0.39 B,b (0.142)

* *p* < 0.05 indicates statistically significant difference.

Averages followed by the same uppercase letter in the column and lowercase letter in the row do not differ at the 5% significance level (*P* < 0.05) by Tukey’s test.

For the Shore A hardness tests, the four-way ANOVA shown in Table 7 highlights a statistically significant difference (*p < 0.001) in time, between time and silicone and between time and pigment used. Further statistical analysis showed that there was an increase in values after 3 months of immersion and washing (T2) for both silicones except for the MDX4-4210 silicone used with the Factor pigment. Indeed, the MDX4-4210 silicone groups with the Factor pigment stood out as having significantly lower Shore A hardness values at both times (T1 and T2) (Table 8).

**Table 7:** Analysis of variance (four-way ANOVA repeated measures) of Shore A hardness values.

Factors	Sum of squares	df	Mean square	*F*	*p*
Time	522.15	1	522.150	455.511	< 0.001
Time × Silicone	135.00	1	135.000	117.771	< 0.001
Time × Pigment	40.02	1	40.017	34.910	< 0.001
Time × Adhesive	3.70	2	1.850	1.614	0.204
Time × Silicone × Pigment	1.07	1	1.067	0.931	0.337
Time × Silicone × Adhesive	6.40	2	3.200	2.792	0.066
Time × Pigment × Adhesive	1.23	2	0.617	0.538	0.585
Time × Silicone × Pigment × Adhesive	4.63	2	2.317	2.021	0.138
Residual	123.80	108	1.146		

**Table 8:** Mean values and standard deviation of Shore A hardness between the different silicones and pigments used.

Silicones	Pigments	Time points	
		Initial (T1)	Final (T2)
A-2186	Factor (Tan FI – 215)	34.9 A,a	37.4 A,b
	Flocking	35.3 A,a	40.6 A,b
MDX4-4210	Factor (Tan FI – 215)	31.8 B,a	32.6 B,a
	Flocking	34.1 A,a	37.2 A,b

* *p* < 0.05 indicates statistically significant difference.

Averages followed by the same uppercase letter in the column and lowercase letter in the row do not differ at the 5% significance level (*P* < 0.05) by Tukey’s test.

(Tukey *p* < 0.05)

## Discussion

Based on the results of this study, the null hypothesis that there would be no statistical difference between color change, Shore A hardness and roughness between silicones MDX4-4210 and A-2186, under the influence of pigments, fixation adhesives and artificial sweat, was rejected.

The main challenge encountered in the performance of a maxillofacial prosthesis is the degradation of its appearance, whether due to changes in color or deterioration of physical properties. The average lifespan of facial prostheses is still only 1–1.5 years, mainly due to the fading of their color [27]. Studies point to the fact that humidity and precipitation have a greater effect on the pigmented silicone elastomer than heat and sun. In addition, human conditions such as sweat and sebum also contribute to the color change of prostheses [28]. It should be noted that there are perceptibility and acceptability thresholds for color differences and color changes in facial prostheses. The study by Lagouvardos et al. demonstrated that changes in luminosity were more noticeable and less accepted by observers than changes in chromatic coordinates (green/red and yellow/blue) [29]. Other authors found in their study that color changes are more perceivable in light skin colors regardless of gender and experience or the observer [30]. According to some authors, ΔE < 1 represents a visually imperceptible color change, values of ΔE ≥ 1.0 represent a visually perceptible color change, with ΔE < 3.3 being a clinically acceptable color change and a ΔE ≥ 3.3 being a clinically unacceptable color change, that is perceptible to untrained observers [31]. In this study, all ΔE values were between 1.43 and 1.9, below 3.3 and above 1, so all groups had a clinically acceptable color change that could be perceived by trained operators [31]. According to the results, the highest color change values were found in the groups that came into contact with adhesive, regardless of the pigmentation used, while the control groups (without adhesive application) had lower ΔE values. In addition, a statistically significant change was found only between the adhesives, which indicates that the presence of pigments in the composition of the adhesives used in this study and the difficulty in completely removing them after application was able to alter the color of the silicone [2]. There are few studies in the literature on adhesives for fixing maxillofacial prostheses and their relationship with color change, so more studies would be needed to corroborate this idea.

To avoid infections, it is necessary to use a chemical disinfection material when handling prostheses, such as washing with neutral pH soap and water. The material used for disinfection must not be aggressive to the tissues and must preserve the properties of silicone [21]. After 3 months of washing with neutral soap and immersion in artificial sweat, the tests carried out indicated a reduction in roughness values in all groups, regardless of the use of adhesives. This can be explained by the continuous polymerization process, which promotes a more complete polymer chain, making the silicone surface smoother over time [21]. Furthermore, when comparing the two silicones, it was observed that the MDX4-4120 silicone had a rougher surface compared to the A-2186 silicone at both times (T1 and T2). This is probably because MDX 4-4210 has a higher concentration of filler in its composition, which promotes greater surface roughness [21]. Despite the expected changes, all the results found in this study are clinically acceptable. According to Goiato et al., the typical clinically acceptable value for average roughness is Ra ≤ 0.8 μm [21].

According to Hatamleh et al. [32], the hardness of silicone elastomers is controlled by the surface characteristics of the polymer network and the density of the cross-linkers. The density of the cross-linkers could affect the length of the polymer chain [33] which results in degradation of the mechanical properties of silicon elastomers in time. It is possible that the increase in hardness of silicones added to pigments with nanoparticles maximizes the network of the silicone matrix, lengthens the polymer chain and leads to an increase in hardness values [34].

After the experimental period, the final Shore A hardness tests showed a statistically significant increase compared to the initial tests, with the exception of the groups with MDX4-4210 silicone and Factor pigment, which differed from the other groups in both tests (T1 and T2) and did not change statistically from the initial to the final time, having lower hardness values. In addition, the average Shore A hardness values were higher for the groups with silicone A-2186 at both times. These results may have been due to the continuous polymerization of the MDX4-4210 and A-2186 [35] silicones, which leads to an increase in their hardness over time, and probably the Factor pigment used in this study may have made it difficult for the MDX4-4210 silicone polymer chains to intertwine, reducing its hardness rate, polymerization [35] and leading to a lower hardness value than the others. This suggests that the MDX4-4210 silicone was influenced by the pigments more than the A-2186 silicone. The hardness values measured before and after the adhesives in our study are consistent with those reported by Sweneey et al. and according to Conroy, the ideal Shore A hardness should be between 25 and 55 [5], studies by Lewis and Castleberry stated that the ideal hardness should be 25–35 [36] units and is acceptable in clinical practice. Our results are between 35.3 and 40.6 shore A. The hardness values found in this study are clinically acceptable.

Despite numerous studies on the physical and mechanical properties of MDX4-4210 and A-2186 silicones, the present study brings a novel contribution by evaluating these silicones under combined clinical conditions, including artificial sweat, different adhesives and two types of pigmentation (including flocking) over a prolonged period of 3 months. The use of flocking fibers is an important innovation in the aesthetic characterization of facial prostheses. Flockings mimic the translucency and texture of human skin, contributing significantly to the natural appearance and patient satisfaction. Despite their clinical relevance, there is a lack of studies analyzing their influence on the physical and optical behavior of silicone prostheses, especially when associated with sweat exposure and adhesive application. Therefore, our findings offer clinically relevant insights that are not yet well-established in the literature. This integrative approach helps guide clinicians in choosing materials and protocols that ensure both durability and aesthetics, improving outcomes in maxillofacial rehabilitation. Furthermore, additional studies would be needed to evaluate the use of patches and artificial sweat using other methods in vitro and vivo.

## Conclusion

The MDX4-4210 and A-2186 silicones, after pigmentation and application of facial adhesives, showed changes in color, Shore A hardness and roughness. Artificial sweat did not influence the results in the present study. Their results were considered clinically acceptable and an excellent option for use in maxillofacial rehabilitation.
